# How Arsenic Poisons

**Published:** 1880-11

**Authors:** 


### HOW ARSENIC POISONS.

Most persons who hold any theory concerning the toxic action of arsenic, still believe the doctrine first propounded by Liebig. Arsenious acid according to this theory, has a great affinity for albuminous substances, and tends to form with them solid compounds. In other words the arsenic coagulates the soluble albumen. In this condition it cannot take part in the vital functions, and hence these functions cease. In this way the so-called corrosive action of arsenic on the coats of the stomach was explained, although it is well known that arsenious oxide has at most only a feeble, caustic action. This theory, which was originally propounded to explain the poisonous action of both arsenious oxide and corrosive sublimate, is wholly false, as far as the former of these is concerned. By experiment it is found that solutions of albumen are not coagulated when treated with arsenious compounds, as they are when treated with corrosive sublimate. In fact, arsenious acid does not have a precipitating effect on any of the liquids of the body, stronger than that of carbonic acid, and yet carbonic acid is not poisonous. Liebig himself, later, saw the fallacy of his theory, and renounced it, without, however, substituting another in its place. The fact that arsenical poisoning, with the usual stomach symptoms, could be produced by dropping arsenical compounds into the eye without hurting the eye itself, was alone fatal to the early Liebigian theory. Late investigations, carried on by Binz and Schulz, (*Archiv fuer Exper. Path. and Pharma.*), have developed a new and much more rational theory. Post mortem investigations have shown that those tissues which specially come in contact with the oxygen of the blood, and utilize it, are those which suffer most in arsenical poisoning. This is especially true of the glandular protoplasm. The neutral salts of arsenic acid are even more poisonous than the corresponding salts of arsenious acid. By the tissues of the body, arsenious acid is oxidized to arsenic acid, and arsenic acid is also reduced to arsenious acid. Any kind of albumen will affect the latter process, while the former is brought about by the albumen of animals and plants only. These facts were proved by experiments. Arsenic acid was reduced, when treated at the temperature of the body, with egg albumen and the fibrin of warm blooded animals. Fresh brain substance had the same effect. The tissues of the pancreas of the liver, and the undecomposed protoplasm of plants, not only reduced arsenic to arsenious acid, but also produced the opposite effect. As a result of a long series of experiments, it is concluded that the destructive effects of arsenical compounds on the system are due to the violent agitation imported by them to the oxygen atoms of the albumen molecules. It is this molecular shock which produces such havoc in the tissues, and this shock is the result of the unnatural activity of the oxygen atoms alternately oxidizing and reducing the arsenical salts. Nitrogen compounds act much in the same way. Indeed the similarity between nitrogen and arsenic extends to many particulars. For instance, nitric oxide is extremely poisonous. It takes oxygen from the tissues, and is converted into hyponitric acid. This latter is a powerfully oxidizing substance. It attacks other tissues, giving up its oxygen and becoming reduced again to nitric oxide. This theory is certainly very ingenious, and the evidence supporting it is good. The authors, indeed, appear to have made out their case. At least the theory which they advance is so far superior to any other, that we feel like accepting it, if not finally, nevertheless provisionally.—*Chicago Medical Review.*

